# Latency of DPOAE in milliseconds and waves

**DOI:** 10.1016/S1808-8694(15)31249-0

**Published:** 2015-10-20

**Authors:** Ualace de Paula Campos, Renata Mota Mamede Carvallo

**Affiliations:** Speech Therapist, Specialized in Clinical Audiology; Full Professor, Associate Professor, FMUSP. Medical School, University of Sao Paulo

**Keywords:** latency of otoacoustic emissions, distortion product - otoacoustic emissions, normal hearing, acoustic stimulation

## Abstract

The latency of Distortion Product Otoacoustic Emissions is defined as the time interval between the wave onset from initial stimulation and the return to the ear canal.

**Aim:**

The aim of this research was to verify the latency of the distortion product otoacoustic emissions in normal hearing adults, analyzing the influence of the gender, ear, frequencies and measurements.

**Study design:**

clinical prospective.

**Material and method:**

The measurements had been taken in milliseconds and waves. It was an experimental study, conducted at São Paulo City in 2003. The sample consisted of 38 adults, 18 men and 20 women. Significant Differences for interactions between frequency, ear, gender and measurements were not observed in relation to the latency in milliseconds and waves. A high correlation between the latency measurements in milliseconds and waves was observed. It was concluded that the latency of distortion product otoacoustic emission diminishes as increases the frequency in milliseconds and the opposite occurs in waves. Statistical differences in latencies of distortion product otoacoustic emission were not observed between gender, ears and measurements.

## INTRODUCTION

Audiological assessment has become increasingly objective and perceptual in the search for abnormalities with the use of information technology advances. The otoacoustic emissions test is an objective, simple and non-invasive procedure that detects even mild hearing losses^1^^,^^2^. Otoacoustic emissions are sounds generated by the cochlea that are recorded in the external acoustic canal. It may be a spontaneous response of the cochlea or some acoustic stimulation^3^.

Emissions occur owing to the movement of the outer hair cells (OHC) and this phenomenon is named electromotility^4^. Electromotility is an active process that allows refined discrimination of audible sounds^5^. It is resulting from the action of efferent auditory pathways in the OHC^6^. Total loss of OHC results in about 60 dB HL of hearing loss^7^.

The emissions are characterized as spontaneous and evoked, and they are the independent occurrence of any stimulus but dependent on presence of a stimulus^8^.

Among evoked emissions, there are three subdivisions: Transient otoacoustic emissions, frequency-stimulus and distortion product. Transient otoacoustic emissions capture short-duration acoustic signals, such as clicks and tone burst. This assessment is useful in the detection of cochlear disorders. Stimulus-frequency evoked otoacoustic emissions are produced by continuous pure tone and show characteristics similar to transient emissions. However, it is not used because of technical difficulties. Distortion product evoked otoacoustic emissions (DPOAE) are two pure tones of different frequencies, presented simultaneously. They represent a non-linear response of the inner ear to pure tone stimuli and consist of new frequencies different from the ones initially presented. They are important given that they analyze the sound frequencies in ranges that vary from 0.5 to 8KHz. The correlation between the two pure tones of distortion product otoacoustic emissions is 1.22 (f2/f1 = 1.22)^9^.

The otoacoustic emissions test captures responses from 98 to 100% of the ears of normal subjects. Depending on the hearing loss, this value is reduced and the losses greater than 30dB HL may not present responses8., 9., 10., 11., 12..

Otoacoustic emissions assess the functional integrity of the cochlea. This exam may be influenced by many different factors, such as sealing of external acoustic canal, middle ear conditions and malformation of external ear^11^.

In 1998, Carvallo et al.^10^ studied the effects of the middle ear on OAEs. The likelihood of capturing DPOAE is 78 times greater in subjects without affection than in those that have them.

DPOAE are evoked by the interaction of two pure tones of different frequencies (f1 and f2). A tertiary tone of a different frequency is produced back to the external auditory canal. f1 is a primary tone with frequency below f2, which is also a primary tone but of a higher frequency. f1/f2 ratio is 1.221,2,8,9.

To understand and find out about the latency of distortion product otoacoustic emissions it is necessary to understand that the progression of the traveling wave through the basilar membrane is dependent on the external hair cells. If latency is determined by the progression of the traveling wave, then the latency measurement will reflect the cochlear function^8^. Mahoney (1993)^13^ defined latency of DPOAE as the time interval in which the wave goes from its initial stimulus and comes back to the external auditory canal. The way it has been used to measure the latency of DPOAE is the method of phase changes^8^^,^^14^. This method consists of variation of frequencies to find latencies. However, the value of latency depends on how f1 and f2 (primary frequencies) are manipulated as a result of frequency^14^.

Previous studies have shown that when we maintain constant f2 (f1-sweep method), the yield of latency of distortion product otoacoustic emissions is 20% less than f2-sweep method, in which f1 is constant^15^.

Latency of distortion product otoacoustic emissions can be measured in two units: milliseconds (ms) and number of waves (w). The number of waves expresses latency as a result of wavelength^16^.

Latencies added to amplitude of DPOAE may provide more complete information to cochlear processes by studying the propagation of traveling wave. It is considered a beneficial tool in investigating the micromechanisms of the cochlea^17^.

Mahoney (1993)^13^ has also described that latency varies depending on frequency, based on the hypothesis that latency is determined by the progression of the traveling wave, related with the theory of frequency cochlear selectivity.

Carvallo and Azevedo (2003)^18^ studied DPOAE in neonates and concluded that there is reduced latency as a result of increased frequency. The authors also observed that there is no mean statistically significant difference comparing right and left ears and there is difference between the genders, but the difference is significant only in one frequency.

Marques and Azevedo (2004)^19^ examined DPOAE in normal hearing subjects and did not find statistically significant difference between the right and left ears and female and male gender.

In a study about high frequency otoacoustic emissions, Dunckley and Dreisbach (2004)^20^ did not find significant differences between male and female subjects, but they considered important to determine what is the effect of gender on DPOAE measurements and how these differences can affect the measurements in clinical use of emissions.

Hoth et al. (2001)^21^, upon analyzing the latency of DPOAE in subjects with hearing loss, concluded that there is no systematic correlation between latency of emissions and hearing loss, indicating that latency of DPOAE does not define the severity of the hearing loss.

Namyslowski et al. (2001)^22^ studied the latency and amplitude of DPOAE in subjects with normal hearing, subjects exposed to noise and elderly patients and found longer mean latencies in subjects exposed to noise and shorter mean latencies in elderly subjects.

Some factors may interfere in the recording of latency of OAE and one of them is spontaneous otoacoustic emissions, which break the linear relation between phase and frequency. However, Wable et al. (1997)^14^ pointed out that this fact is not significant and that men have latencies 16% higher than women, showing the need to analyze the impact of gender on DPOAE.

Bowman, Brown and Kimberley (2000)^23^ stated that the gender differences are attributed to mean differences of cochlear length, because women have the cochlea 13% smaller than men.

In view of the discussion, the objective of the present study was to check latency time (in milliseconds and in number of waves) of distortion product otoacoustic emissions in adults without auditory complaints, analyzing the influence of gender, tested ear, frequency and measurement unit.

## METHOD

This was a prospective and experimental study, performed in the city of Sao Paulo in the year 2003.

### Subjects

We assessed 38 adults without complaint of auditory affection, including 20 women and 18 men, without distinction of race, social-economic-cultural background, residents in Sao Paulo, seen in the Laboratory of Human Hearing Investigations, FMUSP.

The inclusion criteria in the study were:

• Age between 17 and 30 years;
• Absence of external acoustic canal obstruction abnormality;
• Normal thresholds equal to 20dB HL in frequencies of 250 to 8000Hz;
• Type A Tympanogram;
• Presence of ipsilateral acoustic reflexes for stimuli of 500, 1000 and 2000Hz;
• Presence of distortion product otoacoustic emissions in frequencies 2002, 2515, 3174, 4004, 5042 and 6384Hz.

Subjects were volunteers and agreed to participate after they were informed about the procedures.

### Devices

Middle ear analyzer GSI 33 - Grason Stadler Version 2 - Microprocessed and provided with three pure tone probe frequencies for immittance: 226Hz, 678Hz and 1000Hz. The device automatically performs tympanometric measurements at 50 daPa/s and the results are recorded in a graph that is printed by a printer coupled to the system. We used thermo-sensitive paper for printing. The Middle ear analyzer was calibrated for the altitude conditions of the city of Sao Paulo, and all necessary care was taken for the electrical installation, so as to meet the technical specifications of the manufacturer.

Audiometer GSI 61 - Grason Stadler - The device enables performance of audiograms in frequencies of 250 to 20000Hz, in accordance with the following standards: ANSI S3,6-1989, ANSI S3,43-1992, IEC 645-1 (1992), IEC 645-2 (1993), ISSO 389 and UL 544. It has two independent channels, with the accessories for speech audiometry. For conventional audiometry (250 to 8000Hz) phones Telephonics TDH 50ped cse, with impedance of 80ohms, were used.

Cochlear Emission Analyzer ILO 88 v 5.6 and ILO 92 - Otodynamics, London, to perform transient otoacoustic emissions (TOAE) and distortion product otoacoustic emissions (DPOAE) with and without contralateral noise, in addition to distortion product otoacoustic emissions latencies. The device was installed to be used in a acoustic booth, the same as for the audiometer described above, enabling alternate use of both devices.

### Procedures

Subjects were instructed about the purpose of the study in clear and understandable language and signed the free informed consent in the first visit.

First, we performed anamnesis to collect data concerning auditory integrity and meatoscopy to observe the integrity of external acoustic canal. Once the integrity was confirmed, we performed immittanciometry with stimuli of 500, 1000 and 2000Hz ipsilateral, and we considered the responses with variations greater or equal to 0.3mL in admittance of acoustic reflexes to exclude any conductive auditory problem. Audiometry was performed in frequencies of 250, 500, 1000, 2000, 3000, 4000, 6000 and 8000Hz and speech tests (SRT and IPRF), considering thresholds below or equal to 20 dB HL, SRT compatible with frequencies of 500, 1000 and 2000Hz and IPRF with monosyllable words and intelligibility above 88% which were performed to confirm the normal characteristics of the hearing system.

Finally, distortion product otoacoustic emissions were presented for primary frequencies matched in a relation of f2/f1 = 1.2, maintaining the intensity of 70 dB SPL for f1 and 70 dB SPL for f2 (Nf1-Nf2 = 0, where N = intensity of dB SPL). Considering the responses related to signal/noise ratio greater than 3 dB SPL in relation to the second standard deviation of background noise, in frequencies of f2 of 2026, 2563, 3223, 4053, 5139 and 6445Hz. In the study, latency of distortion product otoacoustic emissions was captured by the method of changes in phases, which consists of the variation of frequency to find latencies. We maintain a primary frequency (f1) and another primary frequency (f2) is variable, because the maintenance of the primary frequency f1 as constant (f1-sweep method) ensures yield 20% better than the maintenance of f2 as constant 15. Latency of distortion product otoacoustic emissions was checked in the same frequencies collected in distortion product otoacoustic emissions (2026, 2563, 3223, 4053, 5139 and 6445Hz), in measurements of ms and w, using the latency-gram protocol.

In the analysis of responses, two techniques were used. The statistical technique ANOVA - Analysis of Variance and Person Correlation^24^^,^^25^, adopting the significance level of 0.05 (5%) for rejection of null hypothesis.

## RESULTS

We performed a descriptive analysis of latencies of DPOAE in the two units of measurement (ms and w), separating the right and left ears and male and female gender.

The statistical analysis did not show significant difference between middle ear latency in any of the frequencies (ms and w), despite the fact that in the frequency of 4053Hz values were close to the level of significance in female subjects, both in frequency measurement of ms and w. In female gender, the means of DPOAE latencies in ms were 7.40; 5.96; 4.89; 4.70; 3.66 and 3.12ms for frequencies of 2026; 2563; 3223; 4053; 5139 and 6445Hz, respectively. In male gender, the means of DPOAE latencies in ms were 7.72; 6.76; 5.27; 4.86; 3.72 and 3.47ms for frequencies of 2026; 2563; 3223; 4053; 5139 and 6445Hz.

Latency means of DPOAE in w in female patients were 14.91; 15.14; 15.62; 18.93; 18.67 and 20.07 w for frequencies of 2026; 2563; 3223; 4053; 5139 and 6445Hz, respectively, whereas in male patients, the means of DPOAE latencies were 15.57;17.19; 16.84; 19.58; 18.90 and 22.33 w for frequencies of 2026; 2563; 3223; 4053; 5139 and 6445Hz.

In all comparisons made between the ears, we did not find any statistically significant difference, disregarding the ear effect (Table 1).

**Table 1:** Comparison of latency (in ms) in relation to gender.

Latency		Mean	Median	SD	N	p
2026Hz	M	7,72	7,70	1,84	34	0,571
	F	7,40	7,30	2,75	38	
2563Hz	M	6,76	6,90	1,49	36	0,051#
	F	5,96	5,80	1,95	39	
3223Hz	M	5,27	5,30	1,50	36	0,282
	F	4,89	5,20	1,51	38	
4053Hz	M	4,86	4,75	1,05	36	0,501
	F	4,70	4,50	0,98	39	
5139Hz	M	3,72	3,70	0,70	36	0,725
	F	3,66	3,60	0,75	40	
6445Hz	M	3,47	3,40	1,25	36	0,141

In none of the frequencies there was statistically significant difference between the genders. It is worth mentioning that in frequency of 2563Hz the genders were the same, but there was a tendency to being different. In the analysis in w, we observed the same results, and p-values were 0.559; 0.050; 0.280; 0.490; 0.783 and 0.132 for frequencies of 2026; 2563; 3223; 4053; 5139 and 6445Hz, respectively.

Despite the fact that there were no statistically significant differences between the gender, male ears presented higher latency time in all frequencies, and in 2026Hz, latency of DPOAE was 4.14% greater than in men; in 2563Hz, they were 11.8% greater latency means; in 3223Hz, they were 7.2% greater; in 4053Hz, they were 3.29% greater; in 5139Hz, they were 1.61% greater; in 6445, they were 10.08% greater and in general, latencies of DPOAE in male were 6.35% greater than in female, both in milliseconds and in number of waves.

Given that the difference between male and female genders was irrelevant, it was possible to bring together the two variables. Latencies of DPOAE in milliseconds and number of waves, discarding the differences between gender and ears, are shown in Table 2:

**Table 2:** Means of DPOAE latencies by frequency (in ms and w).

Latency	Measurement Unit	2026Hz	2563Hz	3223Hz	4053Hz	5139Hz	6445Hz
Mean	Ms	7,55	6,34	5,08	4,77	3,69	3,29
	W	15,22	16,12	16,22	19,24	18,78	21,14
Median	ms	7,40	6,50	5,20	4,70	3,70	3,25
	w	14,85	16,50	16,70	18,90	18,75	21,00
Standard	ms	2,36	1,78	1,51	1,01	0,72	1,03
Deviation	w	4,76	4,53	4,84	4,07	3,65	6,52
N		72	75	74	75	76	76
p		< 0,001*					

We noticed that latency (in ms) of all frequencies were different, except for frequencies of 3223Hz and 4053Hz, which were statistically the same.

The results obtained for the measurement unit w were similar with the results of the measurement unit in ms, but we observed that the frequencies of 2026Hz, 2563Hz and 3223Hz were statistically significant different from the other frequencies, which were also different, except between the frequencies of 4053Hz and 5139Hz, which were statistically the same.

The decrease in latencies as a result of increase in frequencies was 16.02% between 2026 and 2563Hz; 19.87% between 2563 and 3223Hz; 6.10% between 3223 and 4053Hz; 22.64% between 4053 and 5139Hz; 10.84% between 5139 and 6445Hz and 56.42% between 2026 and 6445Hz.

**Figure 1:**
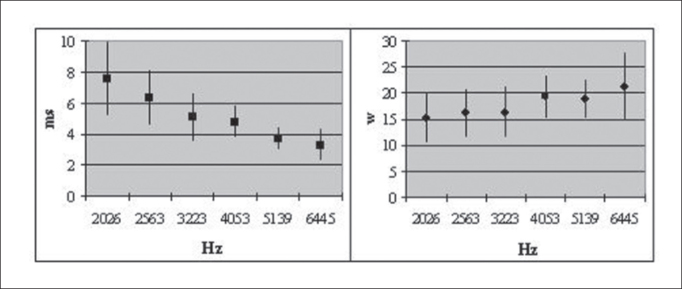
Mean ± 1 SD of latency measurements in ms and w.em w.

The standard deviation for measurements in ms and w presented statistically significant similarity, as we can see visualized in Figure 1.

In the correlation between latencies, in both measurement units (ms and w), we noticed that the measurements of latency presented a significant correlation, that is, latency in milliseconds explained latency in w and/or vice-versa, as presented in Table 3.

**Table 3:** Correlation between latencies in both measurement units (ms and w).

	Correlation	
Frequency		Correlation
Freq. 2026		100,0%
Freq. 2563		100,0%
Freq. 3223		100,0%
Freq. 4053		100,0%
Freq. 5139		99,8%
Freq. 6445		99,3%

## DISCUSSION

The progression of the traveling wave through the basilar membrane is dependent on the outer hair cells and latency is determined by the progression of the traveling wave, that is, latency of DPOAE assesses the cochlear function^8^.

Latencies of DPOAE in ms vary with statistically significant mean, and the value of latency in ms reduces as the frequency increases. Mahoney (1993)^13^ described that latency varied with frequency, based on the hypothesis that latency is determined by progression of the traveling wave, related with the theory of cochlear selectivity of frequency. It is observed that latency was reduced as the frequency increased, because high frequencies are on the base of the cochlea, which makes latency (in ms) of these frequencies lower. Wable (1997)^14^ also found the same data in his study, in addition to observing that latency was decreased as stimulus intensity increased. By comparing latency in different levels of intensity, we can gather information about the cochlear inner processes.

DPOAE latencies, in w, have also varied with statistically significant mean, but the value of w increased with the increase in frequency.

The differences in gender were not significant in any of the measurement units (ms and w), however, the latency in male was on average 6.35% greater than in female patients. In the study performed by Wable et al. (1997)^14^ men had latencies that were 16% greater than women, pointing to the need to analyze the effect of gender in latencies of DPOAE. Bowman, Brown and Kimberley (2000)^22^ stated that there were differences in gender owing to the mean difference of cochlea length, given that women have cochleas that are 13% smaller than men. Carvallo and Azevedo (2003)^18^ have also observed the difference in latency between the genders, but this difference was statistically significant only in frequency of 3000Hz.

There was no mean statistically significant difference between right and left ears, as shown in the study by Marques and Azevedo (2004)^19^, an analysis that disregarded the effect between the ears and analyzed them together.

In the study by Azevedo and Carvallo (2003)^18^, there was also no difference between right and left ears.

The correlation between the two measurement units used in the study was approximately 100% (correlation ≈ 100%). These data are due to the fact that both units assessed the same function. According to the Manual of Otodynamics (1994)^16^, w value represented an alternative form to assess cochlear function. Given that latency in milliseconds has a correlation of approximately 100%. we can say that one variable explains the other and based on these findings, we can have a function in which one measurement unit depends on the other to be found.

The standard deviations of the forms in ms and w did not present any statistically significant difference, showing that any of the measurement units can be used to investigate latency in DPOAE.

The intention of this study, which was initially to better understand the cochlear function, was performed by the analysis in two measurement units (ms and w), contributing with information that had been little studied and that still has to be further investigated. It is expected that the investigation contained in this study may serve as support for further investigations of the topic.

## CONCLUSION

Latencies of DPOAE in ms reduce as frequency of stimulus increases.

Latencies of DPOAE in w increase as frequency of stimulus increases.

We did not observe statistically significant difference in latencies of DPOAE between the ears in ms and w.

We did not observe statistically significant difference in latencies of DPOAE between gender in ms and w, but the measurements of latency of DPOAE in men were greater than the measurements in women.
